# The Vestibular-Evoked Postural Response of Adolescents with Idiopathic Scoliosis Is Altered

**DOI:** 10.1371/journal.pone.0143124

**Published:** 2015-11-18

**Authors:** Jean-Philippe Pialasse, Martin Descarreaux, Pierre Mercier, Jean Blouin, Martin Simoneau

**Affiliations:** 1 Faculté de médecine, Département de kinésiologie, Université Laval, Québec, QC, Canada; 2 Centre de recherche du CHU de Québec, Québec, QC, Canada; 3 Département des sciences de l’activité physique, Université du Québec à Trois-Rivières, Trois-Rivières, QC, Canada; 4 Clinique d’Orthopédie Infantile de Québec, Québec, QC, Canada; 5 Laboratoire de neurosciences cognitives, FR 3C 3512, CNRS—Aix-Marseille Université, Marseille, France; Ludwig-Maximilian University, GERMANY

## Abstract

Adolescent idiopathic scoliosis is a multifactorial disorder including neurological factors. A dysfunction of the sensorimotor networks processing vestibular information could be related to spine deformation. This study investigates whether feed-forward vestibulomotor control or sensory reweighting mechanisms are impaired in adolescent scoliosis patients. Vestibular evoked postural responses were obtained using galvanic vestibular stimulation while participants stood with their eyes closed and head facing forward. Lateral forces under each foot and lateral displacement of the upper body of adolescents with mild (n = 20) or severe (n = 16) spine deformation were compared to those of healthy control adolescents (n = 16). Adolescent idiopathic scoliosis patients demonstrated greater lateral displacement and net lateral forces than controls both during and immediately after vestibular stimulation. Altered sensory reweighting of vestibular and proprioceptive information changed balance control of AIS patients during and after vestibular stimulation. Therefore, scoliosis onset could be related to abnormal sensory reweighting, leading to altered sensorimotor processes.

## Introduction

Spine deformations, such as adolescent idiopathic scoliosis (AIS), represent 90% of all scoliosis. AIS affects 1% to 3% of children in the at-risk population of those aged 10 to 16 years [1]. Despite significant efforts to better understand the aetiopathogenesis of AIS, the cause or sequence of events leading to scoliosis onset are unknown. Nonetheless, it is clear that AIS is a multifactorial disorder involving genetic factors, neurological dysfunctions, hormonal/metabolic dysfunctions, skeletal growth abnormalities, and biochemical factors [2, 3]. How these factors interact in scoliosis onset and whether a combination of factors is involved in curve progression are still unknown.

To control balance, the central nervous system (CNS) receives feedback about the body sways mainly from proprioceptive, visual and vestibular systems; it integrates the signals from the different sensory systems and then generates corrective balance motor commands [4]. One way to study whether AIS patients have sensorimotor impairment is to alter the quality of the sensory information and measure its consequences on balance control. Remarkably, in absence of sensory manipulation, results from various studies have shown that balance control of AIS patients is similar to healthy controls [5–8]. For instance, Adler et al., (1986) observed significantly less body sway on static balance tasks (i.e., two feet together and the tandem Romberg position with the eyes open or closed) for AIS patients than for controls. Furthermore, Herman et al. (1985) measured no difference in body sway between healthy participants and those with adolescent idiopathic scoliosis during quiet stance (i.e., in absence of sensory manipulation). Nonetheless, when sensory information is challenged, results from some studies have demonstrated that balance control of AIS patients is impaired [9, 10]. For example, either during or immediately following ankle proprioception deprivation greater increase in body sway is observed in AIS patients compared to controls supporting the hypothesis that AIS could be related to sensorimotor integration dysfunction [8, 11–14].

The main hypothesis infers that an alteration in sensorimotor integration leads to inappropriate motor commands, resulting in asymmetrical back muscle tone and consequently spine deformation [8]. The vestibular system is one of the structures that may be involved in the onset of scoliosis. This system is involved in torso muscle activation either by direct efferent projections to the spinal cord through the medial vestibulospinal tract or by collateral activity of the reticulospinal tract. Thus, abnormalities in this system could translate into inappropriate phasic or tonic torso muscle activities [15, 16]. If it occurs during a critical period of growth and development, an alteration in such motor commands could be predisposing to the onset of spine deformation [17]. This hypothesis derives from clinical and animal studies demonstrating that stimulation of vestibular nuclei activated the back muscles [18–22].

In addition, results from animal studies suggest that scoliosis can be induced by brain-stem lesions in the early developmental stage [23–25]. The results of clinical studies confirm that some AIS patients have a vestibular impairment [26–28]. For instance, oculomotor and postural vestibular deficits have been reported to range from 50% to 85% in AIS patients, whereas these deficits range from 12% to 17% in controls [16]. Furthermore, these authors reported that a weaker predominance of the right vestibular apparatus is sometimes observed in AIS patients. In addition, the results of a study comparing AIS patients’ ability to process vestibular information to estimate their whole body position following various rotational amplitudes revealed that, compared to the controls, AIS patients underestimated the amplitude of rotations, indicating an impaired ability to process vestibular signals for cognitive purposes [29]. In that study, only AIS patients with severe spine curvature were tested. The authors hypothesized that severe spinal deformity could be partly due to impaired vestibular information travelling from the cerebellum to the vestibular cortical network or an alteration in the cortical mechanisms processing vestibular signals. The latter hypothesis was confirmed in adult xenopus frogs. Unilateral labyrinthectomy at the larval stage resulted in the development of scoliotic deformations due to a permanent asymmetric tone of axial and limb muscles [17, 30]. The authors concluded that any persistent asymmetry in descending pathways during the critical developmental period, regardless of whether it was initiated at the peripheral or central level, could alter the deformable growing skeleton, resulting in scoliotic deformations. In the end, altering the xenopus frogs’ muscle tone caused a severe scoliotic deformation (i.e., Cobb angle > 40°). Overall, the results of these clinical and animal studies confirm that vestibular dysfunction may be related to scoliosis. The question remains, however, whether only AIS patients with severe scoliosis have sensorimotor integration impairments. Therefore, the present work investigates whether vestibulomotor or sensorimotor control is impaired only in AIS with severe scoliotic deformations. It is hypothesized that adolescent with severe idiopathic scoliosis (AIS) would exhibit abnormal vestibular evoked postural responses compared to healthy age-matched individuals and AIS patients with mild spine deformation. If this hypothesis is confirmed, it will suggest that abnormal vestibulomotor response or sensorimotor control may contribute to curve progression. On the other hand, if AIS patients with mild spine deformation also show abnormal vestibular evokes postural response; it will indicate that impaired vestibulomotor or sensorimotor control may be related to scoliosis onset but not necessarily related to curve progression.

## Material and Methods

### Participants

Overall, 52 individuals (41 females and 11 males) were tested. Participants were aged between 10 and 18 years old and were classified in three different groups. Patients with AIS were grouped according to the severity of their scoliotic deformations (i.e., Cobb angle) measured by a paediatric orthopaedic surgeon by means of X-rays. Sixteen participants (mean age: 15.6±1.3 years, mean weight: 61.9±16.2 kg, mean height: 167.2±10.8 cm) with a Cobb angle larger than 30° (mean Cobb angle: 37.3±7.3 degrees) were assigned to the severe AIS group (AIS-S) and 20 participants (mean age: 14.8±1.7 years, mean weight: 54.4±10.4 kg, mean height: 164.9±10.7 cm) with a Cobb angle larger than 15° and smaller than 30° (mean Cobb angle: 20.4±3.6 degrees) made up the mild AIS group (AIS-M). Sixteen healthy participants (mean age: 14.6±2.8 years, mean weight: 52.2±7.5 kg, mean height: 160.6±6.5 cm) with no spine deviation were enrolled in the control group (CTR). Three males were tested in the AIS-M and CTR groups whereas five males were tested in the AIS-S group. Both AIS groups had similar degrees of skeletal maturity as no group difference (t-test, p = 0.54; mean Riser sign: 3.8±1.3 and 4.3±0.7, for AIS-M and AIS-S groups, respectively) was observed for the Risser sign which is an indicator of skeletal maturity ranging from 0 (immature) to 5 (fully mature). The Cobb angle of patients in the AIS-S group was larger than that of participants in the AIS-M group (t-test, p < 0.001). There was no significant difference between the three groups in terms of age or weight (ps > 0.05). Although both AIS groups were of similar height, the AIS-M patients were taller than controls (p < 0.05). Among the scoliosis groups, 6 patients had received conservative treatment (3 in each group) and for 21 patients a body brace had been prescribed (9 and 12, for the AIS-M and AIS-S groups). None of the patients wore a brace during testing. This research study has been approved by Laval University ethics committee. Parents and adolescents gave their written informed consent according to the requirements of Laval University biomedical ethics committee.

### Data recording

Balance control was assessed using two force platforms (AMTI, model BP400600NC-1000, Watertown, MA, USA) and upper body kinematics was recorded using an electromagnetic system (Polhemus–model Liberty 240/8, Colchester, VT, USA). In order to track upper body kinematics, three sensors were attached to each participant; one on the sacrum (L5/S1), one on the seventh cervical vertebra (C7) and one on the forehead (H). A microcontroller (model Basic Stamp BS2px, Parallax, Rocklin, CA, USA) controlled the timing of all equipment. Data were acquired through a digital-to-analogue data acquisition board (model PCI-6224-16 bit, National Instruments, Austin, TX, USA) using custom-made software developed in Matlab (MathWorks, Natick, MA, USA).

### Experimental procedure

Participants were involved in two experimental sessions. The first session served to assess participants’ maximal voluntary lateral flexion. Participants stood upright (one foot on each force platform) with their eyes open and executed three maximal lateral flexion of the trunk on each side. These results allowed us to determine each participant’s spine biomechanical limits. Trunk angle (delimited by the sensors at L5/S1 and C7) relative to the vertical axis was calculated to assess maximal trunk lateral flexion range of motion. Thereafter, for the second experimental session, the feed-forward and feedback control mechanisms was investigated by assessing the postural response to bilateral galvanic vestibular stimulation. Participants stood upright with their head straight ahead, their eyes closed and their feet 2 cm apart, with each foot on a force platform. Each trial was divided in four epochs. The first 2 seconds (baseline balance control epoch [–2 0] s) were used to assess baseline balance control. For the following 2 seconds (vestibular stimulation epoch [0 2] s), galvanic vestibular stimulation (GVS), a step pulse of 1 mA of amplitude lasting 2 seconds, was applied. For 15 trials, the anode was located on the left mastoid process (inducing a right-to-left body movement along the frontal plane), and for 15 trials, the anode was located on the right mastoid process (inducing a left-to-right body movement along the frontal plane). The stimulation side was randomized. GVS were delivered using a DS5 bipolar constant current stimulator (Digitimer Ltd, Garden City, UK). The skin behind the ears over the mastoid processes was prepared using an electrode skin prep pad (Dynarex, Orangeburg, NY, USA) before placing the PALS Platinum 3.2 cm electrodes (Axelgaard Manufacturing Co. Ltd., Fallbrook, CA, USA) bilaterally. The electrodes were secured using 3M Transpore Tape 1527–1 (3M). The first second after GVS offset (post-GVS epoch [2 3] s) enabled us to assess balance control following the cessation of GVS. The following 2 seconds (balance recovery epoch [3 5] s) were used to evaluate whether the participant’s balance control returned to the baseline level. For each trial, the data acquisition started when the participant’s weight was approximately evenly distributed (visual inspection, via an oscilloscope, of the maximum vertical force of each force platform) and head straight ahead (i.e., inter-aural line parallel to the frontal plane). Participants had a rest period every 10 trials.

### Data analysis

To assess postural control, the root mean square (RMS) values of the C7 horizontal displacement was calculated for each interval. To single out balance control resulting from a pure vestibulomotor response versus from the sensorimotor integration of vestibular and proprioceptive information, during the vestibular stimulation epoch, the sum of the lateral force (net lateral force) was studied during two intervals. For instance, the vestibulomotor response (pure feed-forward vestibular reaction) was calculated from the impulse of the net lateral force (i.e., time integration of the net lateral force) from 320 ms to 500 ms after the GVS onset. This period was chosen because it occurred before noticeable movements of the head and torso were observed. Consequently, this initial motor response is probably purely vestibular in origin [31, 32]. The balance control resulting from the sensorimotor integration of vestibular and proprioceptive information was assessed by calculating the RMS value of the net lateral force under each foot for the duration of each interval. The horizontal displacement of C7 and the net lateral force were normalized according to participants’ height and weight, respectively.

It has been reported that some AIS patients have a qualitatively asymmetrical vestibulo-ocular reflex (VOR) gain, suggesting that peripheral vestibular alteration could be impaired in this population. Thus, to control for possible alteration of the vestibular system, the VOR gain was quantified. Participants sat in a completely dark room on a chair located in the centre of a black cylinder with a radius of 1.5 m. The participants were secured to the chair using a four-point belt and a chin support preventing head movement on the trunk during the rotations. The chair was manually rotated around the vertical axis using the handle attached to the rear of the chair. The rotations were measured using an optical encoder (US Digital, model H5S-1024, Vancouver, WA, USA). The amplitude of the chair rotation was either clockwise or counter-clockwise (amplitude of 30° in each direction and six trials per direction). Horizontal eye movements were recorded using electro-oculography (Biomedica Mangoni, model BM623, Pisa, Italy). The participants were instructed to keep their eyes straight ahead throughout the body rotation. Chair rotations and eye movements were recorded at 1000 Hz using a 16-bit A/D board (National Instruments, Model AT-MIO-16DE-10, Austin, TX, USA). For each trial, we calculated the VOR gain as the ratio between the slope of the linear regression from eye and chair positions. The linear regression was fitted using a 75-ms epoch before peak eye velocity. The coefficient of determination (r^2^) was calculated to determine the accuracy of the fitting.

### Statistical analysis

From the mean voluntary maximal lateral flexion of the trunk (first experimental session), a Group (CTR, AIS-M, AIS-S) by Direction (Left, Right) analysis of variance (ANOVA), with repeated measures on the last factor was performed to evaluate whether biomechanical limitations hampered body sway along the frontal plane. To determine whether balance control was influenced by scoliosis or spine curvature severity (second experimental session), Group (CTR, AIS-M, AIS-S) by Interval (baseline balance control [–2 0] s, vestibular stimulation [0 2] s, post-GVS [2 3] s, balance recovery [3 5] s) by Direction (Left, Right) ANOVAs, with repeated measures on the last two factors, were performed for the RMS values of the horizontal displacement of C7 and the net lateral force. Furthermore, to verify whether the feed-forward vestibulomotor response of the two scoliosis groups differed from that of controls, a Group by Direction ANOVA, with repeated measures on the last factor, was performed on the impulse of the net lateral force. To determine whether the scoliosis patients had a different VOR gain than controls, a Group (CTR, AIS-M, AIS-S) by Direction (Left, Right) ANOVA, with repeated measures on the last factor, was performed. According to our hypotheses, a priori contrasts were performed to determine whether either GVS (i.e., change in RMS values from baseline balance control interval [–2 0] s to vestibular stimulation interval [0 2] s) or stopping GVS (i.e., change in RMS values from vestibular stimulation [0 2] s to post-GVS interval [2 3] s) altered the scoliosis groups’ balance control more than that of the controls. For other post hoc comparisons, Tukey tests were used. Finally, to determine if the severity of the spine deformation was related to sensorimotor integration impairment, a Spearman’s rank correlation between spine deformation amplitude (i.e., Cobb’s angle) and RMS value of the net lateral force during the GVS interval was performed.

## Results

### Spine range of motion

The analysis of the maximal voluntary trunk lateral flexion (first experimental session) revealed no main effects of Group or Direction or Group by Direction interaction (ps > 0.05). Group means for maximal trunk angular deviation with respect to the vertical were 35 ± 2°, 39 ± 2° and 40 ± 2°, for the AIS-S, AIS-M and CTR groups, respectively. It should be mentioned that vestibular stimulation induced a lateral trunk flexion of less than 4.6%, 15.1% and 41.4% of the maximal trunk angular displacement for the CTR, AIS-M and AIS-S groups, respectively. Overall, these results confirmed that the amplitude of lateral trunk flexion induced by GVS was unconstrained by either scoliosis or the magnitude of spine deformation.

### Time records of the kinematic and kinetic variables

**Fig 1:**
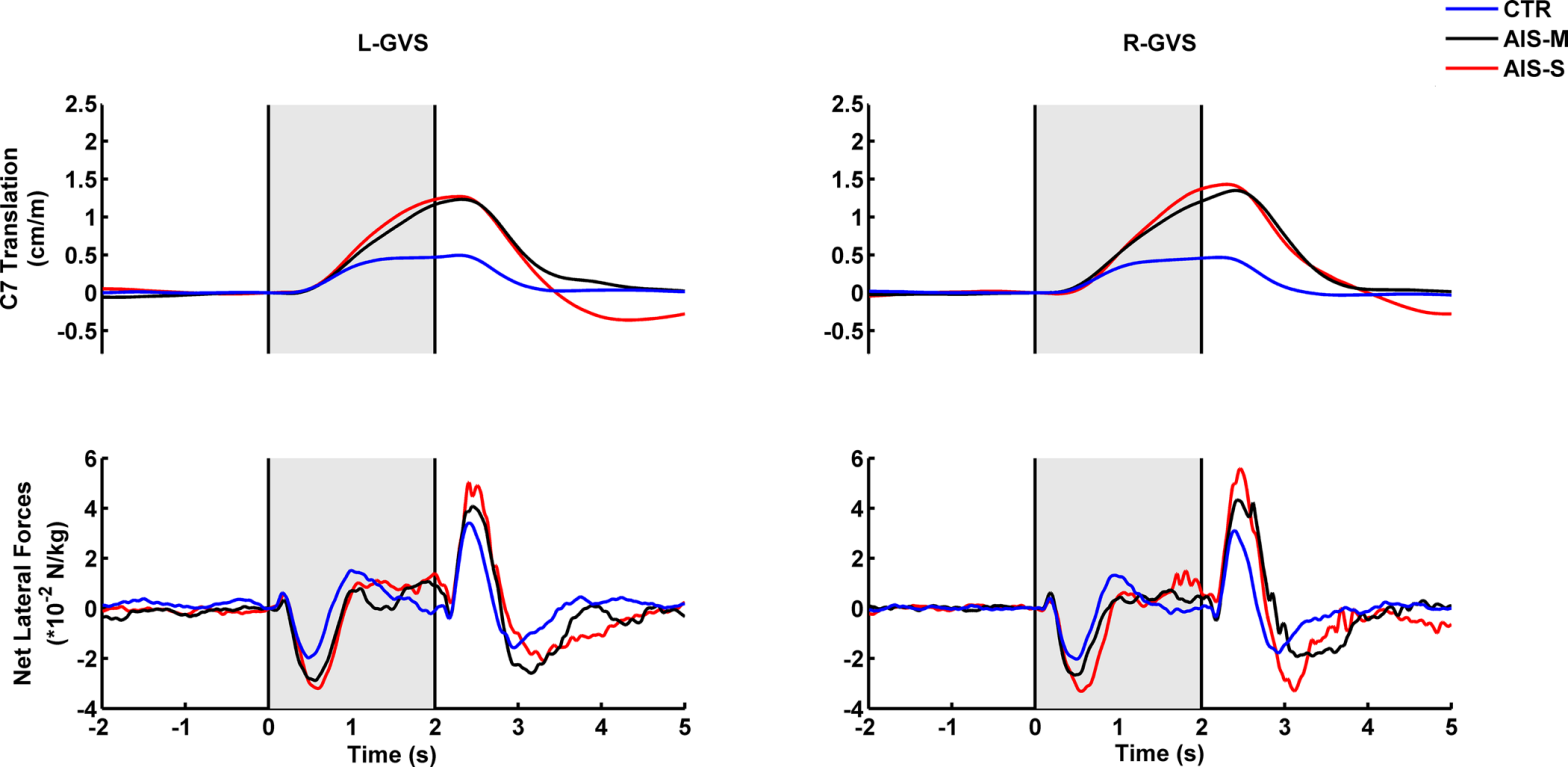
Group mean time-series for C7 horizontal displacement along the frontal plane for left and right vestibular stimulation (left and right upper panels, respectively). Group mean time-series for the net lateral force for left and right vestibular stimulation (left and right lower panels, respectively). The grey areas show the interval of vestibular stimulation. To ease comparison for both GVS directions, the polarity of the time-series is made the same.

Participants reacted to vestibular stimulation by swaying towards the side of the anode (Fig 1, upper panel: to ease comparison between GVS directions, the polarity of the data is identical). The onset of lateral torso displacement occurred approximately 600 ms after vestibular stimulation onset. The postural change resulted from a complex pattern of lateral forces between each foot and the force platforms (Fig 1, lower panel: to ease comparison between GVS directions, the polarity of the data is identical). A small initial peak is observed at approximately 200 ms, followed by a larger opposite peak at approximately 500 ms. The analysis of the feed-forward vestibulomotor response (i.e., 320 to 500 ms following vestibular stimulation onset) concentrates on the larger component of the lateral force because it is responsible for the measured postural change.

### 3.3 Balance Control Resulting from Vestibular and Proprioceptive Information

**Fig 2:**
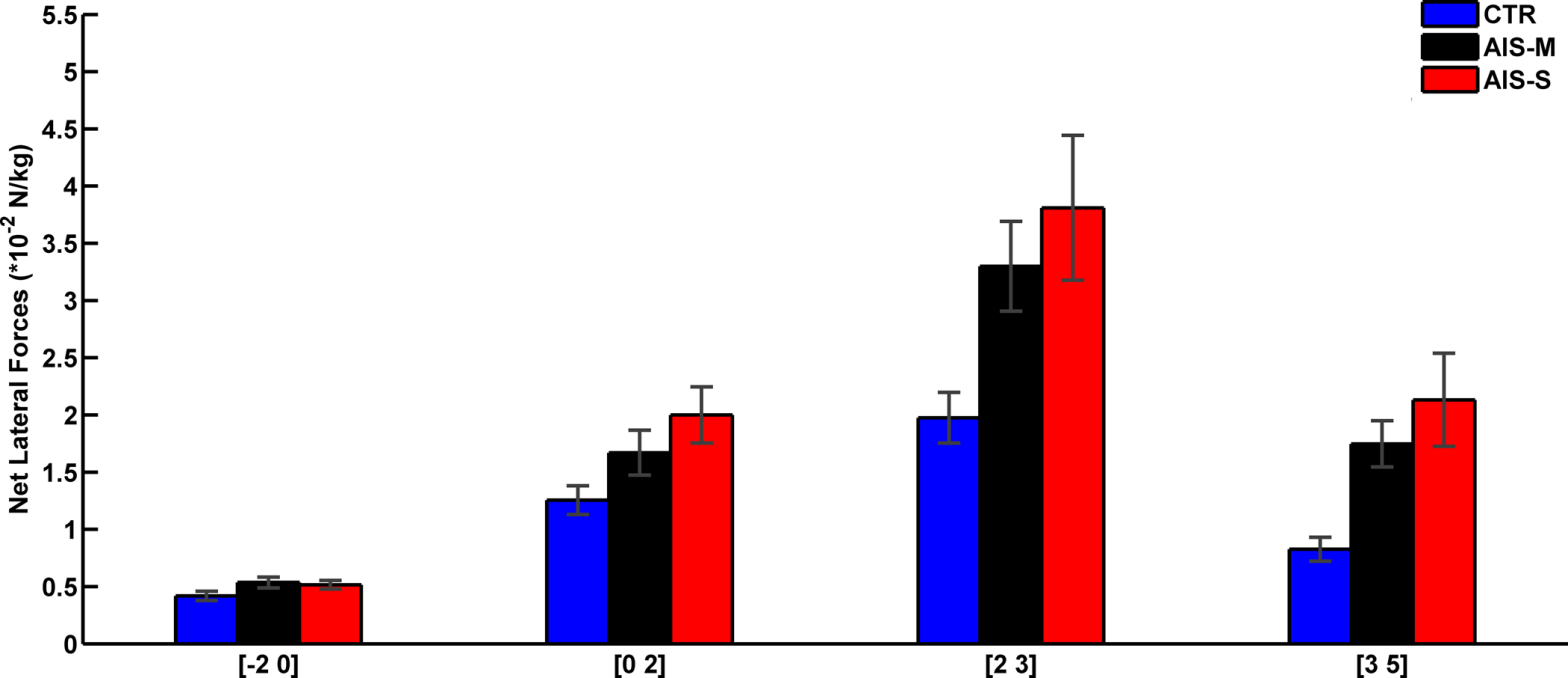
Group means for normalized RMS values of net lateral force for the four intervals. Error bars indicate standard error of the mean.

The analysis of the RMS values of the net lateral forces (Fig 2) demonstrated no main effect of Direction (F(1,49) = 0.03, p = 0.86), but main effects of Interval (F(3,147) = 80.25, p < 0.001) and Group (F(2,49) = 5.24, p < 0.01) and a significant interaction of Group by Interval (F(6,147) = 3.47, p < 0.01). Baseline balance control ([–2 0] s before vestibular stimulation) did not differ across groups; similar RMS values for the net lateral force were observed (p > 0.05). Compared to the baseline control interval, all groups showed larger RMS values (ps < 0.05) during the vestibular stimulation epoch ([0 2] s). The increase in vestibular-evoked balance response, was larger for the AIS-S group than for the CTR group (p < 0.05) but similar to the AIS-M group (p > 0.05). In addition, no difference was observed between the AIS-M and CTR groups (p > 0.05). Stopping the stimulation of the vestibular apparatus (i.e., post-GVS interval [2 3]) altered the balance control (i.e., increase in RMS values) of the AIS-S group while the balance control of the CTR group was unaffected (p < 0.001). Spine deformation severity, however, did not influence balance control following vestibular stimulation (no difference between scoliosis groups, p > 0.05). During the balance recovery interval ([3 5] s, following GVS onset), the RMS values of the net lateral force of the two scoliosis groups did not return to the baseline level ([–2 0] s before GVS onset, p < 0.001). On the other hand, controls did return to their initial balance; their RMS values did not differ from the baseline control interval ([–2 0] s before GVS onset, p > 0.05). Results of the correlation between the spine deformation severity and the RMS value of the net lateral force revealed that the sensorimotor integration impairment was not related to the amplitude of spine deformation (r = 0.08, p > 0.05 and r = -0.02, p > 0.05, for left and right vestibular stimulation, respectively).

### Postural control resulting from vestibular and proprioceptive information

**Fig 3:**
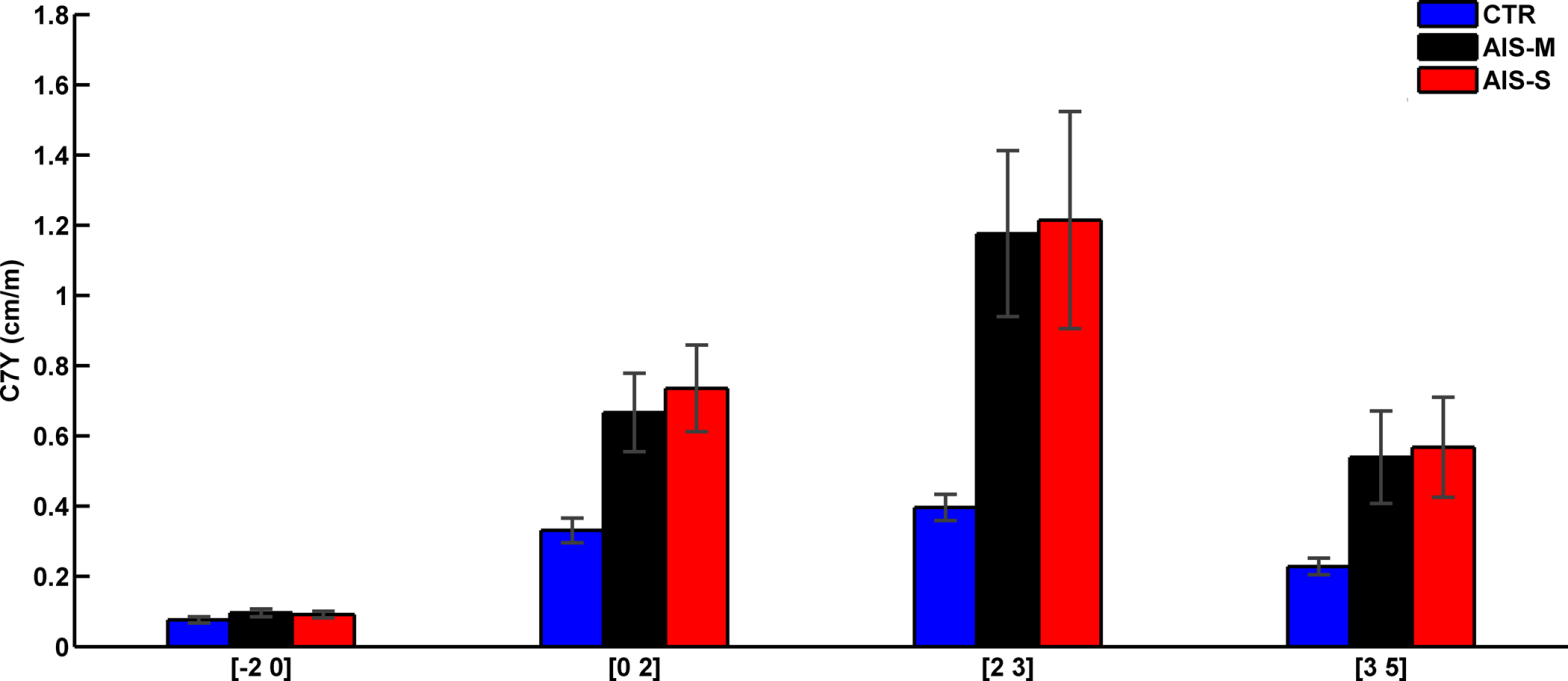
Group means for the RMS values of normalized C7 horizontal displacement along the frontal plane for the four intervals. Error bars indicate standard error of the mean.

The analysis of the horizontal displacement of C7 along the frontal plane (Fig 3) showed no main effect of Direction (F(1,49) = 2.84, p = 0.10), but main effects of Group (F(2,49) = 3.72, p < 0.05) and Interval (F(3,147) = 35.98, p < 0.001) and a significant Group by Interval interaction (F(6,147) = 3.32, p < 0.01). All groups had similar body sway (ps > 0.05) before vestibular stimulation (i.e., baseline balance control interval [–2 0]). Altering the vestibular information increased the body sway of both AIS groups more than in controls (ps < 0.05). Immediately following vestibular stimulation (i.e., post-GVS interval: [2 3]) the body sway of both AIS groups increased more than controls (ps < 0.05). During the balance recovery interval (i.e., [3 5] s following GVS onset), the body sway of the AIS-M group was still greater than during baseline balance control interval (i.e., [–2 0] s before GVS onset, ps < 0.05), and the AIS-S group had the same tendency (p = 0.051). In contrast, the controls succeeded in controlling their balance; their body sway was similar to the baseline balance control interval (i.e., [–2 0] s before GVS onset, p > 0.05).

### Assessment of the vestibulomotor response

We investigated whether the alteration in balance control observed in the AIS groups during vestibular stimulation implied only a dysfunction of the mechanisms transforming vestibular and proprioceptive information into balance motor commands (i.e., sensorimotor integration) or whether it also included an alteration of the feed-forward vestibulomotor response. To do so, the amplitude of the impulse of the net lateral force (i.e., time integration of the net lateral force), before noticeable torso movements (i.e., 320 to 500 ms following GVS onset), was calculated. The analysis of the impulse revealed that both scoliosis groups and controls had similar vestibulomotor responses (no main effects of Group [F(2,49) = 1.30, p > 0.05], Direction [F(1,49) = 1.23, p > 0.05] or Group by Direction interaction [F(2,49) = 0.35, p > 0.05]).

The absence of any group difference for vestibulomotor response is corroborated by the absence of a group difference for the VOR gain measured while participants were seated on the rotary chair. Accordingly, the analysis of the VOR gain (Table 1) revealed no main effect of Group (F(2, 47) = 0.52, p > 0.05) or Direction (F(1, 47) = 2.2, p > 0.05) and no Group by Direction interaction (F(2, 47) = 0.73, p > 0.05). Technical problems prevented us from calculating VOR gain for two of the control participants. The high percentage of variance explained by the linear regression model confirms the efficiency of the model in quantifying the VOR gain.

**Table 1:** Vestibulo-ocular reflex (VOR) gain and variance accounted for (VAF).

	Left rotation		Right rotation	
Group	VORgain	VAF (%)	VORgain	VAF (%)
**Controls**	0.90 (±0.02)	93.0	0.95 (±0.03)	93.0
**AIS-M**	0.92 (±0.02)	94.0	0.92 (±0.03)	94.0
**AIS-S**	0.91 (±0.03)	93.0	0.98 (±0.04)	93.0

## Discussion

The first objective of this study was to assess whether, compared to healthy controls, AIS patients would show an altered feed-forward vestibulomotor response (i.e., 320 ms to 500 ms immediately following vestibular stimulation onset) or sensorimotor integration impairment (during and after the vestibular stimulation; GVS and post-GVS epochs). The second objective was to determine whether such alterations would be observed only in AIS patients with a severe spine deformation or whether sensorimotor impairment scaled with spine deformation. Both AIS groups showed larger body sway than controls during the vestibular stimulation and post-GVS intervals.

During the last decades, few studies have investigated whether AIS patients had abnormal vestibulomotor control. To our knowledge, in those few studies, caloric vestibular stimulation (CVS) was used to evaluate the integrity of the AIS patients’ vestibular apparatus [26, 27]. Although the results from these studies are important, there are differences between caloric and galvanic stimulation of the vestibular apparatus. First, the postural effect of CVS is mediated mainly by the semicircular canals, while little or no otolithic component is involved [33]. Furthermore, the maximal effect of CVS is generally obtained in approximately 80 s and the vestibular sensations can last as long as 15 minutes [34]. Consequently, CVS is not suitable for studying the role of vestibulomotor control in regulating upright balance. In contrast, the onset/cessation effect of GVS is rapid and GVS evokes response in both semicircular canal and otolith afferents [35]. Some studies have indirectly assessed AIS patients’ ability to process vestibular information to control their body sway. When visual and somatosensory systems are challenged simultaneously, AIS patients have larger body sway than controls [5, 10, 11]. In this condition, it is likely that the CNS relies more on vestibular information to control balance, as the reliability of the other sensors is altered. In these studies, during sensory manipulation (e.g., Achilles tendon vibration in absence of vision), vestibular sensory system was mainly the system for sensing body sway. Larger body sway might be explained by a higher variance of vestibular information causing an alteration in reweighting vestibular information.

Vestibular stimulation delivers pure vestibular stimulation that is modulated by somatosensory information from the whole body [36]. For instance, the AIS patients’ body tilt continued for the duration of the stimulus, whereas the controls’ body sways levelled out approximately 1 s after GVS onset (see Fig 2, upper panel). The greater body drift observed in AIS patients could correspond to a motor bias that is corrected in controls because of proper sensory reweighting. It has been suggested that vestibular evoked response could be described as the sum of a step and a ramp response [37]. These authors demonstrated that the position (i.e., step) response results from an effect on otolith afferents, whereas the velocity (i.e., ramp) response arises from an effect on canal afferents. It is worth mentioning that an fMRI study using GVS demonstrated a decrease in the activity of the visual and somatosensory cortex [38]. Therefore, it is possible that a reciprocal inhibitory interaction mechanism permits sensory reweighting through proper processing of proprioceptive or visual information.

The present results suggest that the sensory reweighting mechanism is altered in AIS patients. These data corroborate the results of previous studies. For example, in a study investigating ankle proprioception reintegration in AIS patients [11], it was demonstrated that AIS patients had more difficulty than controls in reweighting ankle proprioceptive information following a brief period of sensory deprivation. In that study, the reweighting of ankle proprioception, whether vision was available or not, led to an increase in the centre of pressure velocity variability (i.e., RMS) in AIS patients with severe spine deformations, whereas the age-matched controls reduced their centre of pressure velocity variability. The present results further extend this observation by demonstrating that a sensory reweighting dysfunction is also present in AIS patients with a moderate spine deformation. The suggestion that there is a central dysfunction is further corroborated by the results of a study investigating the activation of the sensorimotor network in AIS patients [14]. Those authors found that, when participants performed a motor task using the right or left hand, the BOLD signal in the contralateral supplementary motor area (SMA) of AIS patients was larger than in controls. The over-activation of the SMA was interpreted as evidence of a sensorimotor transformation dysfunction in the AIS patients, since the SMA is involved in sensory integration and sends direct projections to the primary motor cortex and the spinal cord. It is possible that the central mechanism comparing semicircular canal information with somatosensory information from the plantar sole and lower limbs is altered, explaining why the body sway kept increasing during the GVS, as also observed in the deafferented patient [36]. Nonetheless, it is unclear whether the AIS patients were unable to attenuate the gain of the semicircular canals (i.e., the slope of the ramp response; [37].

Greater weighting of the vestibular feedback information to control body sway might be related to the integrity of the somatosensory system [36, 39]. Nevertheless, we doubt that the AIS patients had an impaired lower limb proprioceptive threshold, because they demonstrated similar balance stability to controls during the baseline interval. Furthermore, measurements of nerve conduction velocity in the fibular and median nerves did not support the hypothesis that polyneuropathy is related to idiopathic scoliosis [40]. The fact that the VOR gain and the impulse evoked by the net lateral force (i.e., pure vestibulomotor control) were similar in the scoliosis groups and controls suggest that the vestibular end organ, vestibular nerve or vestibular brain-stem nucleus of AIS patients were not malfunctioning. However, a study using VEMPs at different levels of the spinal cord is needed to further assess the integrity of the vestibulo-spinal system in AIS patients. Previous studies also did not find peripheral vestibular dysfunction in their AIS patients [28, 29, 41]. Consequently, the present experimental results support the suggestion that a dysfunction in the sensory reweighting mechanisms led to an altered descending motor commands (i.e., balance motor commands).

To explain the fact that, regardless of spine deformation magnitude, both AIS groups had a sensory reweighting impairment, one could suggest that the spine deformation of the mild AIS patients might progress over time. According to Lonstein and Carlson [42], the incidence of curve progression is related to the magnitude of the curve, chronological age and skeletal maturity. Therefore, to verify this possibility, we determined if skeletal maturity (Risser sign) was related to larger RMS values either during or after vestibular stimulation. Note that a patient with a Risser sign of 1 has a 26% incidence of curve progression, whereas a patient with a Risser sign equal to or greater than 2 has approximately a 10% incidence of curve progression. The result of the Pearson correlation between the Risser sign and the RMS values of the net lateral force revealed no relationship (ps > 0.05, mean r^2^ < 0.05). Only six AIS patients (four mild and two severe) had a Risser sign smaller than or equal to 3, and only one AIS-M patient had Risser sign of 1. The mean Risser sign did not differ between the two scoliosis groups; therefore, it is unlikely that mild spine deformation will progress over time.

## Conclusion

The present data extend previous findings by demonstrating that, both during and after vestibular stimulation, the balance control of AIS patients with a mild or a severe spine deformation is altered compared to controls. Consequently, scoliosis onset could be related to abnormal sensory reweighting leading to altered sensorimotor transformation. It remains to be determined whether sensorimotor transformation dysfunction occurs before curve progression. Furthermore, it is necessary to develop tools to rapidly identify AIS patients with abnormal sensorimotor control. Such tools could become early biomarkers.
